# Outcomes of Combined Heart-Kidney Transplantation in Older Recipients

**DOI:** 10.1155/2023/4528828

**Published:** 2023-06-24

**Authors:** Curry Sherard, Vineeth Sama, Jennie H. Kwon, Khaled Shorbaji, Lauren V. Huckaby, Brett A. Welch, Chakradhari Inampudi, Ryan J. Tedford, Arman Kilic

**Affiliations:** ^1^College of Medicine, Medical University of South Carolina, Charleston, SC, USA; ^2^Division of Cardiothoracic Surgery, Medical University of South Carolina, Charleston, SC, USA; ^3^Department of Surgery, University of Pittsburgh Medical Center, Pittsburgh, PA, USA; ^4^Department of Cardiology, Medical University of South Carolina, Charleston, SC, USA

## Abstract

**Objectives:**

The upper limit of recipient age for combined heart-kidney transplantation (HKT) remains controversial. This study evaluated the outcomes of HKT in patients aged ≥65 years.

**Methods:**

The United Network of Organ Sharing (UNOS) was used to identify patients undergoing HKT from 2005 to 2021. Patients were stratified by age at transplantation: <65 and ≥ 65 years. The primary outcome was one-year mortality. Secondary outcomes included 90-day and 5-year mortality, postoperative new-onset dialysis, postoperative stroke, acute rejection prior to discharge, and rejection within one-year of HKT. Survival was compared using Kaplan–Meier analysis, and risk adjustment for mortality was performed using Cox proportional hazards modeling.

**Results:**

HKT in recipients aged ≥65 significantly increased from 5.6% of all recipients in 2005 to 23.7% in 2021 (*p*=0.002). Of 2,022 HKT patients in the study period, 372 (18.40%) were aged ≥65. Older recipients were more likely to be male and white, and fewer required dialysis prior to HKT. There were no differences between cohorts in unadjusted 90-day, 1-year, or 5-year survival in Kaplan–Meier analysis. These findings persisted after risk-adjustment, with an adjusted hazard for one-year mortality for age ≥65 of 0.91 (95% CI (0.63–1.29), *p*=0.572). As a continuous variable, increasing age was not associated with one-year mortality (HR 1.01 (95% CI (1.00–1.02), *p*=0.236) per year). Patients aged ≥65 more frequently required new-onset dialysis prior to discharge (11.56% vs. 7.82%, *p*=0.051). Stroke and rejection rates were comparable.

**Conclusion:**

Combined HKT is increasing in older recipients, and advanced age ≥65 should not preclude HKT.

## 1. Introduction

Rates of combined heart-kidney transplantations (HKTs) have increased dramatically over the last decade and at a higher rate than isolated heart transplantation (HT) [1–3]. This increase has been driven in part by coexisting renal disease among heart transplant recipients, which has demonstrated a negative impact on survival following isolated HT [3–11]. In addition, recent studies have confirmed the survival benefit of HKT in patients with coexisting heart and renal failure. Importantly, the demand for donor hearts continues to prolong waitlist times for isolated HT and increase the number of patients using mechanical circulatory support, increasing the likelihood of second-organ failure [2, 12–15]. Kidney dysfunction affects the majority of patients awaiting HT, and severe dysfunction may not be reversible after isolated HT, necessitating consideration of HKT in these patients [2, 14].

Advanced age is associated with worse posttransplant survival in isolated heart and isolated kidney transplants, but the relationship between age and post-HKT survival has not been elucidated [5]. However, due to the lack of clear selection criteria, many centers consider advanced age 65 years and older as a contraindication for combined heart-kidney transplantation. There are limited data regarding outcomes among older patients who undergo HKT. The aim of this study was to evaluate trends and outcomes of HKT in patients aged 65 years or older compared to younger patients.

## 2. Materials and Methods

### 2.1. Study Design

The United Network for Organ Sharing (UNOS) is a database that records all solid organ transplantations performed in the United States. The UNOS database was queried for all HKTs performed between January 1, 2005, and June 1, 2021. Only patients over 18 years of age were included in the analysis. This study was deemed exempt from review by the Medical University of South Carolina Institutional Review Board.

Patients were grouped by age, either <65 years or ≥65 years at the time of transplantation. Baseline characteristics of recipients, donors, and recipient-donor matching were compared between the two age groups. The primary outcome was one-year mortality after HKT. Secondary outcomes included 90-day and 5-year mortality, postoperative new-onset dialysis, postoperative stroke, acute rejection prior to discharge, and rejection within one-year of HKT.

### 2.2. Statistical Analysis

Categorical variables are summarized using counts and percentages. Pearson's chi-square tests were used to compare categorical variables, and Fisher's exact tests were used if the frequency of any variable was <5. All continuous variables were nonparametrically distributed and are presented as medians and interquartile ranges. Continuous variables were compared using Kruskal–Wallis tests.

The Kaplan–Meier analysis was utilized to model 1-year survival, which was compared using log-rank tests and Wilcoxon-Breslow-Gehan tests. Multivariable Cox proportional hazards modeling was utilized to calculate the risk-adjusted hazard for recipient age on 1-year mortality after HKT. Covariates associated with 1-year mortality on univariable analysis with *p* < 0.20 were included in the final multivariable model as well as those retained after backward stepwise selection with *p* < 0.05. The threshold for statistical significance was two-sided *p* < 0.05. Analyses were performed using Stata, version 16.1 (StataCorp, TX, USA).

## 3. Results

### 3.1. Patient Cohort

**Figure 1:**
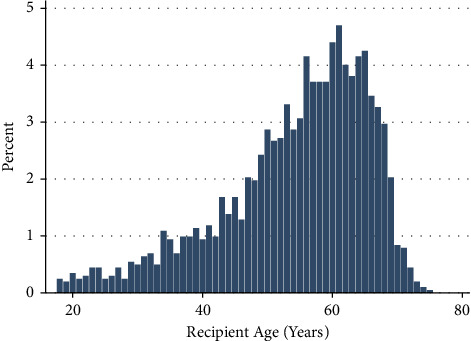
Age distribution of patients undergoing combined heart-kidney transplantation.

**Figure 2:**
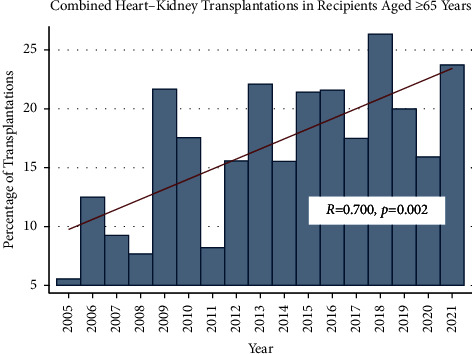
Annual frequency of combined heart and kidney transplantation among recipients aged ≥65 years.

In the observed study period, 2,022 patients underwent HKT. The median age of the study population was 57 years, with 372 (18.4%) aged greater than 65 years. The age distribution of patients undergoing combined HKT is shown in Figure 1. There was a significant increase in the annual frequency of HKT in recipients aged 65 years and older from 5.6% of all HKT recipients in 2005 to 23.7% of all recipients in 2021 (*p*=0.002) (Figure 2).

### 3.2. Baseline Characteristics of the Study Population

Demographic characteristics for HKT recipients stratified by age are summarized in Table 1. Notable characteristics associated with HKT recipients older than 65 included male sex (85.75% vs. 76.30%, *p* < 0.001), white race (61.83% vs. 48.97%, *p* < 0.001), lower creatinine (median Cr 2.2 vs. 2.7, *p* < 0.001), less dialysis prior to HKT (30.28% vs. 52.76%, *p* < 0.001), and fewer waitlist days (median 57.5 vs. 74.5, *p* = 0.039).

Demographic characteristics for HKT donors to recipients aged ≥65 years included higher donor age (median 32 vs. 30, *p* < 0.001), Hispanic ethnicity (24.80% vs. 20.22%, *p*=0.014), and less HLA-matching at ≥3 loci (10.38% vs. 15.27%, *p*=0.016).

### 3.3. Kaplan–Meier Survival after Heart-Kidney Transplantation

**Figure 3:**
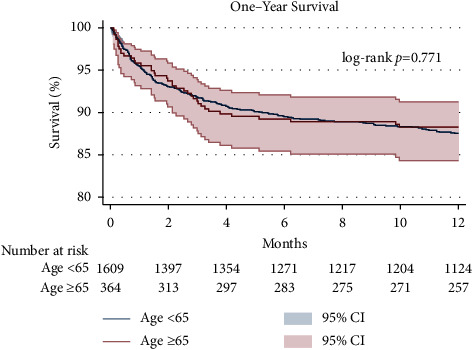
Kaplan–Meier analysis of one-year survival after combined heart and kidney transplantation stratified by recipient age.

**Figure 4:**
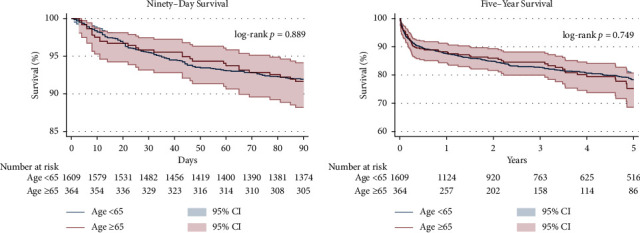
Kaplan–Meier analysis of 90-day and 5-year survival after combined heart and kidney transplantation stratified by recipient age.

A Kaplan–Meier analysis of one-year survival after combined HKT stratified by recipient age is shown in Figure 3. The Kaplan–Meier analysis of 90-day and 5-year survival after combined HKT stratified by recipient age is shown in Figure 4. There were no differences between age groups in 90-day (92.0% in recipients aged <65 years vs. 91.7% in recipients aged ≥65 years, log-rank *p*=0.889), 1-year (87.5% vs. 88.2%, log-rank *p*=0.771, Breslow *p*=0.821), or 5-year (77.8% vs. 76.0%, log-rank *p*=0.748, Breslow *p*=0.992) survival in unadjusted Kaplan–Meier analysis. Secondary outcomes included dialysis prior to discharge (7.82% in younger patients vs. 11.56% in older patients, *p*=0.051), stroke prior to discharge (3.35% vs. 2.20%, *p*=0.375), rejection prior to discharge (8.30% vs. 9.68%, *p*=0.392), and rejection treated within one year posttransplant (8.66% vs. 7.92%, *p*=0.700).

### 3.4. One-Year Survival following Heart-Kidney Transplantation

A multivariable Cox proportional hazards model for one-year mortality following combined HKT with age as a categorical variable is shown in Table 2. After risk adjustment, age ≥65 years was not associated with an increased risk for one-year mortality (HR 0.91, 95% CI, 0.63–1.29, *p* = 0.572). A multivariable Cox proportional hazards model for one-year mortality following combined HKT with age as a continuous variable is shown in Table 3. Increasing age was not associated with an increased risk for one-year mortality after HKT (HR 1.01 per year, 95% CI 1.00–1.02, *p* = 0.236). Increasing body mass index (BMI), increasing serum creatinine and bilirubin at time of HKT, mechanical ventilation prior to HKT, increasing heart ischemic time, and increasing donor age were found to independently predict one-year mortality after HKT.

### 3.5. Secondary Outcomes after Heart-Kidney Transplantation

Secondary outcomes after combined HKT stratified by age are shown in Table 4. Patients aged ≥65 years had higher rates of new-onset dialysis after HKT prior to discharge, though this relationship was not significant (11.56% vs. 7.82%, *p*=0.051). There were no significant differences in rates of stroke prior to discharge, rejection prior to discharge, and rejection treated within one-year posttransplant between age groups.

## 4. Discussion

Currently, there are few clearly defined recipient criteria for combined HKT and little data to suggest which recipients may benefit most from HKT compared to isolated HT [3, 4]. Previous studies have explored the impacts of comorbidities, age, and other recipient characteristics on survival post-HKT in an attempt to generate guidelines for dual organ allocation [5, 6, 13, 16, 17]. Advanced age in particular is a well-described independent risk factor for early and late mortality after isolated HT, although its effect on HKT is less defined. Early outcomes, including 1-year survival, freedom from rejection at 1-year, and absence of major adverse cardiac events in HKT recipients aged ≥65 years, have been shown to be similar to outcomes of isolated HT in this patient group [5]. In addition, analyses of longer term outcomes found that 5-year and 15-year survival after HKT among patients aged ≥60 years was comparable to younger patients [6, 13, 16]. Other factors studied include a threshold eGFR to recommend combined HKT versus isolated HT, with the conclusion that HKT should be recommended in patients with an eGFR <37 mL/minute as it improves posttransplant survival in this group as compared with isolated HT [1]. LVAD implantation has been shown to transiently improve eGFR in patients awaiting heart transplantation, but this effect is temporary and has no impact on survival [18]. Therefore, HKT offers a survival benefit to select patients with cardiorenal disease, which may extend to those of advanced age if selected appropriately. Peripheral vascular disease, recipient age >65 years, nonischemic heart failure, dialysis at the time of HKT, and mechanical circulatory support have previously been identified as factors associated with reduced survival following HKT [17]. The findings presented in this analysis demonstrate similar early posttransplant outcomes among recipients aged ≥65 years compared to their younger counterparts.

These findings corroborate previous research that shows the lack of an adverse impact of advanced age on HKT outcomes. While one study by Reich et al. found that recipient age greater than 65 was associated with worse survival, the majority of previous studies found no difference in survival following HKT based on recipient age [5, 6, 13, 16, 17]. The similar survival rates between younger and older patients in these studies may be attributed to several factors. In a recent analysis by Punnoose et al., recipient selection appeared to mitigate any potential negative impact of advanced age on post-HKT survival [16]. Older patients had fewer severe comorbidities than younger patients but higher incidences of ischemic cardiomyopathy, and younger patients more frequently had risk factors such as smoking, dialysis dependence prior to transplant, mechanical circulatory support prior to transplant, and increased pulmonary artery pressure [16]. These differences illustrate the importance of comorbidities in predicting HKT outcomes rather than age alone. Another contributing factor to the similar overall outcomes of older patients could be related to the rates of graft rejection. Several studies have previously shown that older heart transplant recipients have lower rates of rejection and associated complications than younger recipients due to aging-related deterioration of the natural immune response [19, 20]. Aging has been associated with fewer alloreactive T cells and an increased susceptibility to immunosuppressive agents, producing a reduced rejection rate [20]. This study found that advanced recipient age was not associated with a similar incidence of rejection compared to age <65 years.

Currently, there is a lack of guidelines regarding an upper limit of recipient age for HKT and minimal literature regarding this subject. The findings of the present study are consistent with the available literature on this matter, which recommend an individualized approach to HKT patient selection rather than a defined age cut-off [5]. Reich et al. recommended evaluation of factors, such as BMI, diabetic glycemic control, severe cerebral or peripheral vascular disease, frailty, social support, and severe cognitive-behavioral disabilities [5]. Additionally, Schaffer et al. described dialysis dependence in patients awaiting HT as an indication for HKT as opposed to isolated HT [15]. Given the findings of the present study that mechanical ventilation prior to HKT, increasing heart ischemic time, and increasing donor age independently predict 1-year mortality post HKT; these factors should also be considered in recipient evaluation and donor selection.

Limitations of this study include selection bias as recipients aged 65 years or older were more likely to be of male sex, white race, and have lower creatinine, representing favorable risk characteristics at baseline. Following risk-adjustment, however, recipient age had no impact on increased risk for one-year mortality when modeled as a continuous or categorical variable. In addition, this analysis considered posttransplant complications only occurring before discharge and rejection occurring only within 1-year of transplant. Complications occurring after 1-year posttransplant are not captured by this analysis as these data are not widely available in the UNOS registry. Therefore, differences in longitudinal outcomes other than mortality between age groups are not analyzed here. Furthermore, registry data do not capture center-level practice differences between programs performing HKT. It is possible that only high volume, experienced centers are performing HKT in older patients, leading to improved outcomes and further contributing to selection bias. Lastly, the registry does not contain granular information on patient-specific factors such as perioperative care and postoperative transplant management that could impact survival.

This analysis of the UNOS registry determined that advanced age ≥65 is not predictive of mortality after HKT. Data collected from this cohort indicate that recipient aged ≥65 years is associated with similar one- and five-year survival following HKT as compared with younger recipients. While comorbidities and other factors that are more common in older age may lead to negative outcomes, advanced age alone should not be used as an excluding variable for HKT candidacy.

## Figures and Tables

**Table 1:** Demographic characteristics of patients undergoing heart-kidney transplantation stratified by age.

	Age <65	Age ≥65	*p* value
	*N* = 1,650	*N* = 372	
	81.60%	18.40%	
Recipient			
Age (years), median (IQR)	54 (46, 60)	67 (66, 68)	<0.001
Male sex, no. (%)	1,259 (76.30)	319 (85.75)	<0.001
Race/ethnicity, no. (%)			<0.001
White	808 (48.97)	230 (61.83)	
Black	589 (35.70)	91 (24.46)	
Hispanic	146 (8.85)	32 (8.60)	
Other	107 (6.48)	19 (5.11)	
BMI (kg/m^2^), mean (SD)	26.5 (23.0, 30.3)	26.3 (23.9, 29.2)	0.916
Creatinine (mg/dL), median (IQR)	2.7 (1.9, 4.3)	2.2 (1.7, 3.1)	<0.001
Dialysis prior to transplant, no. (%)	851 (52.76)	109 (30.28)	<0.001
Total bilirubin (mg/dL), median (IQR)	0.7 (0.5, 1.1)	0.7 (0.5, 1.2)	0.293
Diabetes, no. (%)	716 (43.39)	173 (46.51)	0.275
Heart failure etiology, no. (%)			<0.001
Nonischemic cardiomyopathy	623 (37.76)	101 (27.15)	
Ischemic cardiomyopathy	592 (35.88)	208 (55.91)	
Hypertrophic/restrictive cardiomyopathy	105 (6.36)	36 (9.68)	
Failed OHT	252 (15.27)	17 (4.57)	
Congenital heart disease	31 (1.88)	1 (0.27)	
Other/unknown	47 (2.85)	9 (2.42)	
ICU at time of transplant, no. (%)	753 (46.54)	170 (46.70)	0.955
Mechanical ventilation, no. (%)	16 (0.97)	8 (2.15)	0.057
Bridging method			0.186
None	429 (26.00)	99 (26.61)	
Inotropes	486 (29.45)	107 (28.76)	
IABP	212 (12.85)	64 (17.20)	
Durable VAD	414 (25.09)	82 (22.04)	
Temporary VAD/ECMO	109 (6.61)	20 (5.38)	
Karnofsky index, no. (%)			0.316
≥80%	138 (8.94)	24 (6.72)	
50–70%	346 (22.41)	88 (24.65)	
≤40%	1,060 (68.65)	245 (68.63)	
Cardiac index (L/min/m^2^), median (IQR)	2.39 (1.94, 2.91)	2.36 (1.94, 2.90)	0.615
Mean PAP (mmHg), median (IQR)	30 (23, 37)	28 (23, 36)	0.121
Days on waitlist, median (IQR)	74.5 (22, 235)	57.5 (18, 185)	0.039
Heart ischemic time (hours), median (IQR)	32 (25, 38)	32 (25, 38)	0.703
Donor			
Age (years), median (IQR)	30 (22, 39)	32 (24, 43)	<0.001
Male sex, no. (%)	1,195 (72.42)	278 (74.73)	0.366
Race, no. (%)			0.014
White	1,026 (62.87)	224 (60.38)	
Black	240 (14.71)	40 (10.78)	
Hispanic	330 (20.22)	92 (24.80)	
Other	36 (2.21)	15 (4.04)	
Mechanism of death, no. (%)			0.506
Trauma	803 (48.67)	167 (44.89)	
Cerebrovascular	310 (18.79)	73 (19.62)	
Drug overdose	246 (14.91)	65 (17.47)	
Other	291 (17.64)	67 (18.01)	
BMI (kg/m^2^), mean (SD)	26.5 (23.3, 30.3)	26.2 (23.4, 30.3)	0.952
Diabetes, no. (%)	40 (2.44)	10 (2.72)	0.754
Recipient-donor matching			
Sex-matched, no. (%)	1,242 (75.27)	283 (76.08)	0.745
Race-matched, no. (%)	679 (41.15)	116 (44.62)	0.220
HLA-matched, no. (%)^b^	248 (15.27)	39 (10.38)	0.016
ABO-identical, no. (%)	1,389 (84.18)	306 (82.26)	0.363
CMV-matched, no. (%)^c^	837 (50.73)	197 (52.96)	0.437

^a^Donor and recipient are considered HLA-matched if there are fewer than 4 mismatched loci. ^b^Any combination other than CMV D-/R+. BMI, body mass index; CMV, cytomegalovirus; ECMO, extracorporeal membrane oxygenation; HLA, human leukocyte antigen; IABP, intraaortic balloon pump; PAP, pulmonary artery pressure; VAD, ventricular assist device.

**Table 2:** Multivariable Cox proportional hazards model for one-year mortality following combined heart and kidney transplantation.

	Univariable analysis		Final multivariable model	
	Hazard ratio (95% CI)	*p* value	Hazard ratio (95% CI)	*p* value
Age ≥65	0.95 (0.68–1.34)	0.771	0.91 (0.63–1.29)	0.572
Female recipient	1.11 (0.82–1.52)	0.476		
Recipient race/ethnicity				
White	Reference	Reference		
Black	0.89 (0.66–1.19)	0.423		
Hispanic	0.75 (0.44–1.26)	0.271		
Other	1.16 (0.70–1.93)	0.562		
Recipient BMI (per kg/m^2^)	1.04 (1.01–1.06)	0.006		
Creatinine (per mg/dL)	1.04 (0.99–1.09)	0.110	1.05 (1.00–1.11)	0.035
Dialysis prior to transplant	1.32 (1.02–1.71)	0.038		
Total bilirubin (per mg/dL)	1.03 (1.01–1.05)	0.002	1.03 (1.01–1.05)	0.002
Recipient diabetes	1.22 (0.94–1.57)	0.140		
Cardiac diagnosis				
NICM	Reference	Reference		
Ischemic cardiomyopathy	1.17 (0.87–1.57)	0.303		
HCM/RCM	1.20 (0.72–2.00)	0.490		
Failed OHT	0.76 (0.48–1.22)	0.259		
Congenital heart disease	1.20 (0.44–3.29)	0.717		
Other/unknown	0.97 (0.42–2.22)	0.940		
ICU at the time of transplantation	1.15 (0.89–1.50)	0.281		
Mechanical ventilation at the time of transplantation	3.48 (1.72–7.04)	0.001	2.90 (1.33–6.31)	0.007
Bridging method				
None	Reference	Reference	Reference	Reference
Inotropes	0.67 (0.46–0.97)	0.033	0.78 (0.53–1.15)	0.206
IABP	0.95 (0.61–1.47)	0.811	1.07 (0.68–1.69)	0.759
Durable VAD	1.10 (0.78–1.56)	0.578	1.26 (0.87–1.83)	0.220
Temporary VAD/ECMO	1.44 (0.87–2.39)	0.156	1.44 (0.82–2.52)	0.203
Karnofsky index, no. (%)				
≥80%	Reference	Reference		
50–70%	1.58 (0.86–2.89)	0.142		
≤40%	1.55 (0.88–2.73)	0.129		
Cardiac index (per L/min/m^2^)	1.00 (0.83–1.19)	0.965		
Mean PAP (per mmHg)	1.03 (1.01–1.04)	<0.001		
Waitlist time (per day)	1.00 (1.00–1.00)	0.171		
Ischemic time (per hour)	1.19 (1.06–1.33)	0.003	1.17 (1.04–1.31)	0.007
Donor age (per year)	1.01 (1.00–1.03)	0.011	1.02 (1.00–1.03)	0.007
Donor race/ethnicity				
White	Reference	Reference		
Black	1.11 (0.77–1.61)	0.574		
Hispanic	0.86 (0.61–1.22)	0.399		
Other	1.77 (0.93–3.35)	0.082		
Mechanism of death				
Trauma	Reference	Reference		
Cerebrovascular	1.35 (0.96–1.88)	0.082		
Drug overdose	1.09 (0.73–1.63)	0.657		
Other	1.27 (0.89–1.80)	0.187		
Donor BMI (per kg/m^2^)	1.01 (0.99–1.03)	0.277		
Donor diabetes	1.07 (0.48–2.42)	0.861		
Sex-matched	1.04 (0.77–1.40)	0.781		
Race-matched	1.08 (0.83–1.40)	0.583		
HLA-matched	1.28 (0.86, 1.95)	0.210		
ABO-identical	1.06 (0.75, 1.49)	0.755		

BMI, body mass index; CMV, cytomegalovirus; ECMO, extracorporeal membrane oxygenation; HLA, human leukocyte antigen; IABP, intraaortic balloon pump; PAP, pulmonary artery pressure; VAD, ventricular assist device. Age as categorical variable.

**Table 3:** Multivariable Cox proportional hazards model for one-year mortality following combined heart and kidney transplantation.

	Univariable analysis		Final multivariable model	
	Hazard ratio (95% CI)	*p* value	Hazard ratio (95% CI)	*p* value
Age (per year)	1.01 (1.00–1.02)	0.262	1.01 (1.00–1.02)	0.236
Creatinine (per mg/dL)	1.04 (0.99–1.09)	0.110	1.06 (1.01–1.12)	0.014
Total bilirubin (per mg/dL)	1.03 (1.01–1.05)	0.002	1.03 (1.01–1.05)	0.002
Mechanical ventilation at time of transplantation	3.48 (1.72–7.04)	0.001	2.85 (1.31–6.22)	0.008
Bridging method				
None	Reference	Reference	Reference	Reference
Inotropes	0.67 (0.46–0.97)	0.033	0.78 (0.53–1.15)	0.210
IABP	0.95 (0.61–1.47)	0.811	1.06 (0.67–1.66)	0.807
Durable VAD	1.10 (0.78–1.56)	0.578	1.26 (0.87–1.83)	0.872
Temporary VAD/ECMO	1.44 (0.87–2.39)	0.156	1.49 (0.85–2.62)	0.852
Ischemic time (per hour)	1.19 (1.06–1.33)	0.003	1.17 (1.04–1.32)	0.007
Donor age (per year)	1.01 (1.00–1.03)	0.011	1.02 (1.00–1.03)	0.012

ECMO, extracorporeal membrane oxygenation; IABP, intraaortic balloon pump; VAD, ventricular assist device. Age as continuous variable. Only age and covariates associated with one-year mortality on univariable analysis are listed here.

**Table 4:** Secondary outcomes after combined heart-kidney transplantation stratified by age group.

	Age <65	Age ≥65	*p* value
	*N* = 1,650	*N* = 372	
	81.60%	18.40%	
Dialysis prior to discharge	129 (7.82)	43 (11.56)	0.051
Stroke prior to discharge	54 (3.35)	8 (2.20)	0.375
Rejection prior to discharge	137 (8.30)	36 (9.68)	0.392
Rejection treated within one year posttransplant	100 (8.66)	21 (7.92)	0.700

## Data Availability

The data that support the findings of this study are available on request to the United Network of Organ Sharing (UNOS) database.
